# Comparative Analysis of Commercially Available Typhoid Point-of-Care Tests: Results of a Prospective and Hybrid Retrospective Multicenter Diagnostic Accuracy Study in Kenya and Pakistan

**DOI:** 10.1128/jcm.01000-22

**Published:** 2022-11-30

**Authors:** Jyotshna Sapkota, Rumina Hasan, Robert Onsare, Sonia Arafah, Sam Kariuki, Sadia Shakoor, Farah Qamar, Sheillah Mundalo, Frida Njeru, Rael Too, Elizabeth Ndegwa, Jason R. Andrews, Sabine Dittrich

**Affiliations:** a FIND—the Global Alliance for Diagnostics, Geneva, Switzerland; b Department of Microbiology, Nepal Medical College, Kathmandu, Nepal; c The Special Programme for Research and Training in Tropical Diseases, World Health Organization, Geneva, Switzerland; d Aga Khan University, Karachi City, Pakistan; e Faculty of Infectious and Tropical Diseases, London School of Hygiene and Tropical Medicine, London, United Kingdom; f Centre for Microbiology Research, Kenyan Medical Research Institute, Nairobi, Kenya; g Division of Infectious Diseases, Massachusetts General Hospital, Boston, Massachusetts, USA; h Nuffield Department of Medicine, University of Oxford, Oxford, United Kingdom; Medical College of Wisconsin

**Keywords:** blood culture, point-of-care tests, rapid diagnostic tests, *Salmonella* Typhi, typhoid, blood culture

## Abstract

Blood and bone marrow cultures are considered the gold standard for the diagnosis of typhoid, but these methods require infrastructure and skilled staff that are not always available in low- and middle-income countries where typhoid is endemic. The objective of the study is to evaluate the sensitivity and specificity of nine commercially available Salmonella Typhi rapid diagnostic tests (RDTs) using blood culture as a reference standard in a multicenter study. This was a prospective and retrospective multicenter diagnostic accuracy study conducted in two geographically distant areas where typhoid is endemic (Pakistan and Kenya; NCT04801602). Nine RDTs were evaluated, including the Widal test. Point estimates for sensitivity and specificity were calculated using the Wilson method. Latent class analyses were performed using R to address the imperfect gold standard. A total of 531 serum samples were evaluated (264 blood culture positive; 267 blood culture negative). The sensitivity of RDTs varied widely (range, 0 to 78.8%), with the best overall performance shown by Enterocheck WB (72.7% sensitivity, 86.5% specificity). In latent class modeling, CTK IgG was found to have the highest sensitivity (79.1%), while the highest overall accuracy was observed with Enterocheck (73.8% sensitivity, 94.5% specificity). All commercially available Salmonella Typhi RDTs evaluated in the study had sensitivity and specificity values that fell below the required levels to be recommended for an accurate diagnosis. There were minimal differences in RDT performances between regions of endemicity. These findings highlight the clear need for new and more-accurate Salmonella Typhi tests.

## INTRODUCTION

Typhoid fever is an enteric bacterial infection caused by the bacterium Salmonella enterica serovar Typhi (S. Typhi), which is primarily transmitted through contaminated food or water (1). Symptoms include prolonged fever, fatigue, headache, nausea, abdominal pain, constipation or diarrhea, with severe cases leading to serious complications and even death. In 2018, it was reported that 11 to 20 million people worldwide contract typhoid each year, resulting in 128,000 to 161,000 deaths (2).

Because of the primary mode of transmission, typhoid cases are most prevalent in places with poor sanitation and a lack of safe drinking water—most commonly low- and middle-income countries (LMICs) (2). Of these, the regions of endemicity with the highest incidence of typhoid are sub-Saharan Africa (accounting for 40% of all cases), South Asia, North Africa, and the Middle East (2). While the disease can be effectively treated with antibiotics, escalating global antimicrobial resistance, including the emergence of extensively drug-resistant S. Typhi in Pakistan and azithromycin-resistant S. Typhi in multiple countries, indicates that short- to medium-term control of typhoid through vaccination may be the best strategy for reducing disease burden in endemic populations (3, 4). However, with no reliable point-of-care diagnostic, accurately measuring disease burden in order to effectively target areas where routine vaccination would provide the greatest benefit remains a challenge (4).

An accurate diagnosis of typhoid can prove difficult, since the characteristic symptoms are similar to other undifferentiated febrile illnesses such as malaria or dengue fever, and typhoid can also be mistaken for vector borne febrile illnesses such as scrub typhus (5). As such, microbiological testing is usually required to confirm a diagnosis of S. Typhi, with blood and bone marrow cultures currently considered the gold-standard tests (6, 7). However, blood culture testing can be expensive, has a low sensitivity, and requires infrastructure and skilled staff that are not always available in LMICs and are not adequate for rapid patient management (6, 7). Tests using bone marrow cultures may have a higher sensitivity than blood culture tests, but they are not routinely performed as obtaining bone marrow aspirates involves skilled invasive techniques (6, 7). As a result, alternative tests have been widely adopted, especially in LMICs. The most widely used rapid diagnostic test (RDT) is the Widal test, despite numerous reports of poor sensitivity and specificity, which in some cases has led to multiple misdiagnoses in disease outbreaks, treatment delays, and even deaths (6, 8–10). Limitations have also been reported for other typhoid RDTs, such as Typhidot (Reszon Diagnostics), Tubex (IDL Biotech), and Test-it Typhoid IgM (LifeAssay Diagnostics) (6). While these show improvements over the Widal test, they still only exhibit moderate sensitivity and specificity (6). However, many accuracy studies into the effectiveness of typhoid RDTs have variations in methodology and reference standards, making it difficult for health care providers and policymakers to make robust decisions or recommendations for the utility of commercial tests in different settings.

The primary aim of this study was to evaluate the sensitivity and specificity of various commercially available typhoid RDTs using the same protocol in different endemic settings and comparing against a single reference standard available in regions of endemicity. In parallel, we aimed (i) to develop a biorepository of well-characterized specimens for use in evaluating emerging diagnostic tests for typhoid and for supporting future test development and (ii) to evaluate the operational characteristics (including invalid and indeterminate testing rates) of the RDTs.

## MATERIALS AND METHODS

### Study design.

This was a prospective and retrospective multicenter diagnostic accuracy study to evaluate the sensitivity and specificity of commercially available typhoid RDTs, using blood culture testing as a reference standard (NCT04801602). The study was conducted in accordance with the principles of the Declaration of Helsinki, the International Conference on Harmonization of technical requirements for registration of pharmaceuticals for human use (Good Clinical Practice guidelines—ICH-GCP E6 [R2]), and all applicable local IRBs and national laws and regulations. An ethics review board in Pakistan (AKUH Ethics Review Committee) and Kenya (KEMRI Scientific and Ethics Review Unit, National Commission for Science Technology and Innovation and Pharmacy and Poison Board) approved the protocol and study forms. The study was conducted across nine study sites in two geographically distant regions of typhoid endemicity (Pakistan [South Asia] and Kenya [sub-Saharan Africa]) between October 2020 and July 2021. Prospective patients were recruited upon clinical encounters at one of the clinical study sites. Retrospective blood culture samples (that had been tested for S. Typhi) were collected from a previous population-based infectious disease study (SSC 2761, Population-Based Infectious Disease Surveillance [PBIDS]: a platform to measure disease burden and evaluate the impact of public health interventions in Asembo, Siaya County and Kibera, Nairobi County, Kenya) to ensure an adequate sample size for analysis. The aim of the study was to establish a PBIDS platform suitable for tracking causes and burden of common infectious diseases such as typhoid fever over time in both urban and rural settings.

### Patients.

**(i) Prospective enrollment.** Patients eligible for prospective inclusion in the study were 2 to 65 years of age (8 to 65 years in Kenya), presenting with fever or an axillary temperature of >37.5°C (or a history of 3 or more days of fever in the week prior to enrollment), a clinical suspicion of enteric fever, and a body weight >8 kg, who had been admitted to hospital within the past 12 h or had presented to the outpatient department or emergency department of the participating study site. Patients were enrolled in three hospitals in Kenya from February to July 2021 and from October 2020 to July 2021 in Pakistan from six health centers. Prior to enrollment written informed consent were obtained from all patients. For participants younger than 18 years, consents were provided by their guardian. In addition to parental consent, additional written consents were obtained from children aged 12 to 17 years. Upon enrollment, clinical officers asked questions regarding symptoms, history of typhoid, other medical illnesses, treatment history, and status of typhoid vaccination.

Patients were excluded from the study if they were unable or unwilling to provide the required blood samples for analysis, were receiving anticoagulant medications, did not provide informed consent, or had a known noninfectious or non-typhoid-related cause of fever.

**(ii) Retrospective enrollment.** Retrospective serum samples (from study SSC 2761) included in the study were taken from patients with fever of <7 days duration with a minimum volume of 0.5 mL. Samples were nonhemolytic, had <3 freeze thaw cycles and had been stored at or below −20°C. From the parent study (SSC 2761), all residents of the two study sites—Asembo and Kibera, Kenya—were included for household surveillance upon consent from the household head. For the health facility (clinic) surveillance at St. Elizabeth Lwak Mission Hospital in Asembo and Tabitha Medical Clinic in Kibera, patients who were residents of the respective PBIDS areas were included in the study if they visited the facility and fulfilled the case definition of the target infectious diseases (11). The study population in the retrospective study included individuals of all age groups who had experienced acute febrile illness (1 week) with an axillary temperature of >38°C during data collection.

### Investigational products and study procedures.

Blood samples were obtained from each patient upon enrollment in the study by trained phlebotomists using venipuncture and then transported to a central laboratory within 24 h (Aga Khan University in Pakistan; Centre for Microbiology Research [CMR] at the Kenyan Medical Research Institute [KEMRI] in Kenya), and blood culture was carried out. Blood culture is the standard of care for both Pakistan and Kenya for diagnosis of fever. S. Typhi RDTs were carried out once blood culture results were available; samples were stored at −20°C or below until analyzed. Readers of RDTs were blinded to the results of the blood culture tests and *vice versa*.

After testing, any remaining serum samples were aliquoted and stored at −80°C for future evaluation of diagnostic tests (via the https://www.finddx.org/biobank-services/specimen-bank/mrf/).

**(i) Rapid diagnostic tests.** For RDTs, 4- and 3-mL blood samples were drawn from adult patients and children, respectively. After transportation to the central laboratories, the serum was separated, and RDTs were performed according to the manufacturer’s instructions by trained laboratory personnel.

Nine typhoid RDTs were selected for use in the study based on their quality (must be CE-marked), published data, international availability, required sample volume, cost, and turnaround time (see Table S1 in the supplemental material). Of the nine tests, one measured the presence of S. Typhi and/or *S.* Paratyphi antigens (Diaquick *S.* Typhi/Paratyphi Ag cassette [DIALAB]; only used in Pakistan), four measured immunoglobulin G (IgG) and IgM anti*-S.* Typhi antibodies (SD Bioline *S.* Typhi IgG/IgM Fast [Abbott]; Typhidot Rapid IgG/IgM combo test [Reszon Diagnostics International]; Typhoid IgG/IgM Combo Rapid Test CE [CTK Biotech]; and Typhoid IgG/IgM Rapid Test Cassette [Spectrum Diagnostics]), and three measured just the IgM antibodies (TUBEX TF [IDL Biotech]; Enterocheck WB [Tulip diagnostics]; Test-it Typhoid IgM [Life Assay]). The Widal test, which measures the presence of “O” and “H” anti*-S.* Typhi and *S.* Paratyphi antibodies, was also included due to its widespread use in regions of endemicity. RDTs were performed by two trained technicians in both countries. The testing flow of RDTs is available in Fig. S1.

**(ii) Blood culture tests.** For blood culture tests, 10- and 3.5-mL blood samples were drawn from adult patients and children, respectively. After transportation to the central laboratories, all blood samples were tested using established automated systems: BactAlert in Pakistan and BD Bactec 9050 FX 200 in Kenya. When a positive signal was identified by the automated systems, the samples were subcultured in MacConkey agar and blood agar plates. Non-lactose-fermenting colonies on MacConkey agar were biochemically identified for S. Typhi and confirmed by Salmonella-specific antisera. (12) Retrospective samples were included which were identified by similar methods. For the purposes of this study, patients were classified as typhoid or nontyphoid cases according to the blood culture result. Serum samples from other isolates, including *S.* Paratyphi A, were not included in the study.

**(iii) Study team training.** All of the technicians performing RDTs were trained on the RDT procedure based on the manufacturer’s instructions. Two technicians from each site were trained on the RDT procedure. Videos of each RDT procedure were provided to the study team.

### Sample size and statistical analysis.

Based on the reported prevalence of S. Typhi in the regions of endemicity (7% in Pakistan; 5% in Kenya [based on hospital data]) and assuming a low sensitivity and specificity for the RDTs (50% for each), it was estimated that sample sizes of 2,900 patients in Pakistan and 2,000 patients in Kenya would be required. This would ensure 200 positive cases and 200 negative cases in Pakistan and 100 positive cases and 100 negative cases in Kenya, enabling the estimation of RDT sensitivity with 80% power to detect 95% confidence intervals (CIs) of ±10% in Pakistan and ±14% in Kenya (Wilson method). The retrospective analysis of samples was planned to ensure that these targets were met in the event of recruitment difficulties.

The main analysis population was the modified intent-to-test (mITT) population, defined as all patients who were enrolled in the study (intent-to-test [ITT] population) and for whom blood culture test results and at least one RDT test result was available.

Patient characteristics are presented descriptively. RDT sensitivity and specificity values were defined using true-positive (TP), true-negative (TN), false-positive (FP), and false-negative (FN) rates, which were calculated using S. Typhi blood culture results as the reference standard. Sensitivity was defined as TP/(TP + FN), and specificity was defined as TN/(FP + TN). Point estimates for sensitivity and specificity with 95% CIs were calculated using the Wilson method.

Because blood culture has modest sensitivity (estimated at ~60%) to diagnose typhoid, we perform Bayesian latent class modeling to estimate sensitivity and specificity of the RDTs. This approach leverages prior information about the accuracy of blood culture, along with the joint results of all the RDTs, for estimating each RDT’s accuracy. For tests that had both IgG and IgM results, we selected the better-performing isotype from the crude analysis for the latent class analysis. Following previously published methods (13), we defined sensitivity and specificity of each diagnostic by a Beta distribution and used a Gibbs sampler to jointly estimate the sensitivity and specificity, as well as the prevalence of typhoid. We used 100,000 Monte Carlo iterations, discarded the first 50,000, and thinned by 100, reporting median posterior estimates with 95% credible intervals (CrI). We used Beta distributions with α and β equal to 1 for RDT sensitivity, specificity, and typhoid prevalence, α and β equal to 57 and 38 for blood culture sensitivity (60% [95% CI = 50 to 70%]), and a presumed specificity of >99.99%. Latent class analyses were performed using base R and coded by authors.

### Data availability.

We confirm that the data used to support the findings of this study are all available within the article.

## RESULTS

### Study population and patient characteristics.

**FIG 1:**
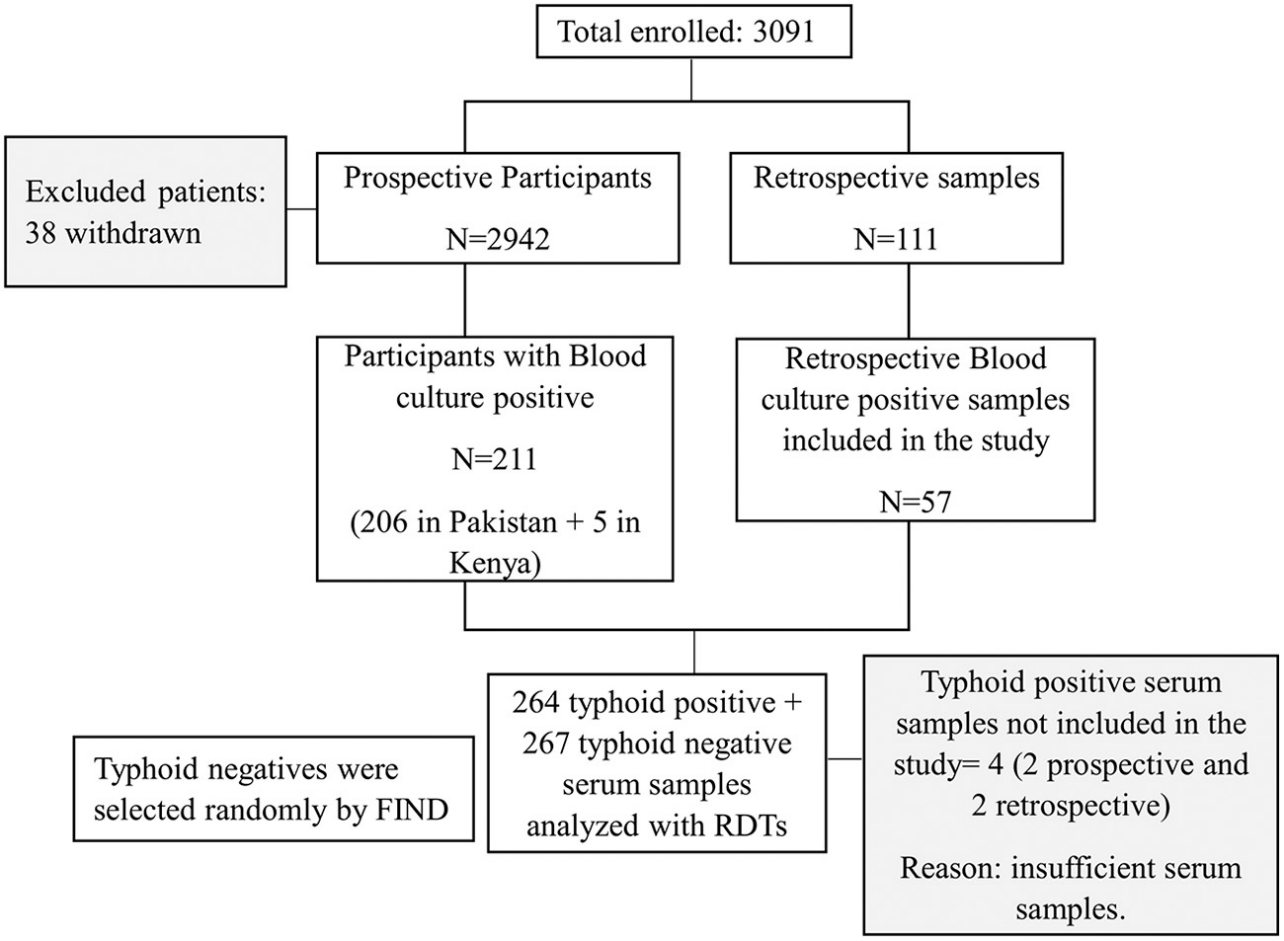
Patient disposition. RDT, S. Typhi rapid diagnostic test.

Patient enrollment and selection is presented in Fig. 1. Overall, 3091 patients were enrolled in the study (2,980 prospectively and 111 retrospectively), of whom 38, who refused blood collection after having given consent, were withdrawn. A total of 2,942 blood culture samples were collected prospectively and 111 samples retrospectively. Of these, 268 were positive for S. Typhi (211 from prospective samples and 57 from retrospective samples). Of 2,942 samples collected prospectively, 255 were contaminated. Other isolates were identified as *S.* Paratyphi A, Escherichia coli, Staphylococcus aureus, coagulase-negative staphylococcus, Pseudomonas spp., and Acinetobacter spp.

Patient characteristics (ITT population) are summarized in Table 1. Although most patients were >17 years of age (58%), the large majority of positive S. Typhi cases (71.1%) were identified in children of <12 years. Among the retrospective samples, the majority of positive cases were also identified in children of <12 years (31/57). The proportion of patients who had received a typhoid vaccination was low (4.8% overall), despite an ongoing vaccination campaign in Pakistan.

**TABLE 1:** Patient characteristics, including S. Typhi prevalence (ITT population)

Parameter	Total		Typhoid positive		Typhoid negative	
	No.	%	No.	%	No.	%
Total blood culture	2,687	100	211	7.9	2,476	92.1
Pakistan	2,155	100	206	9.6	1,949	90.4
Kenya	532	100	5	0.9	527	99.1
Age (mean = 22.7)	2,687		211		2,476	
<12 yrs	900	33.5	150	71.1	750	30.3
12–17 yrs	229	8.5	10	4.7	219	8.8
>17 yrs	1,558	58	51	24.2	1,507	60.9
Single-dose TCV received^*a*^	144	4.8	15	7.1	114	4.6

a A typhoid vaccination campaign was ongoing in Pakistan during the study. ITT, intent-to-test; TCV, typhoid conjugate vaccine.

### RDT sensitivity and specificity.

**FIG 2:**
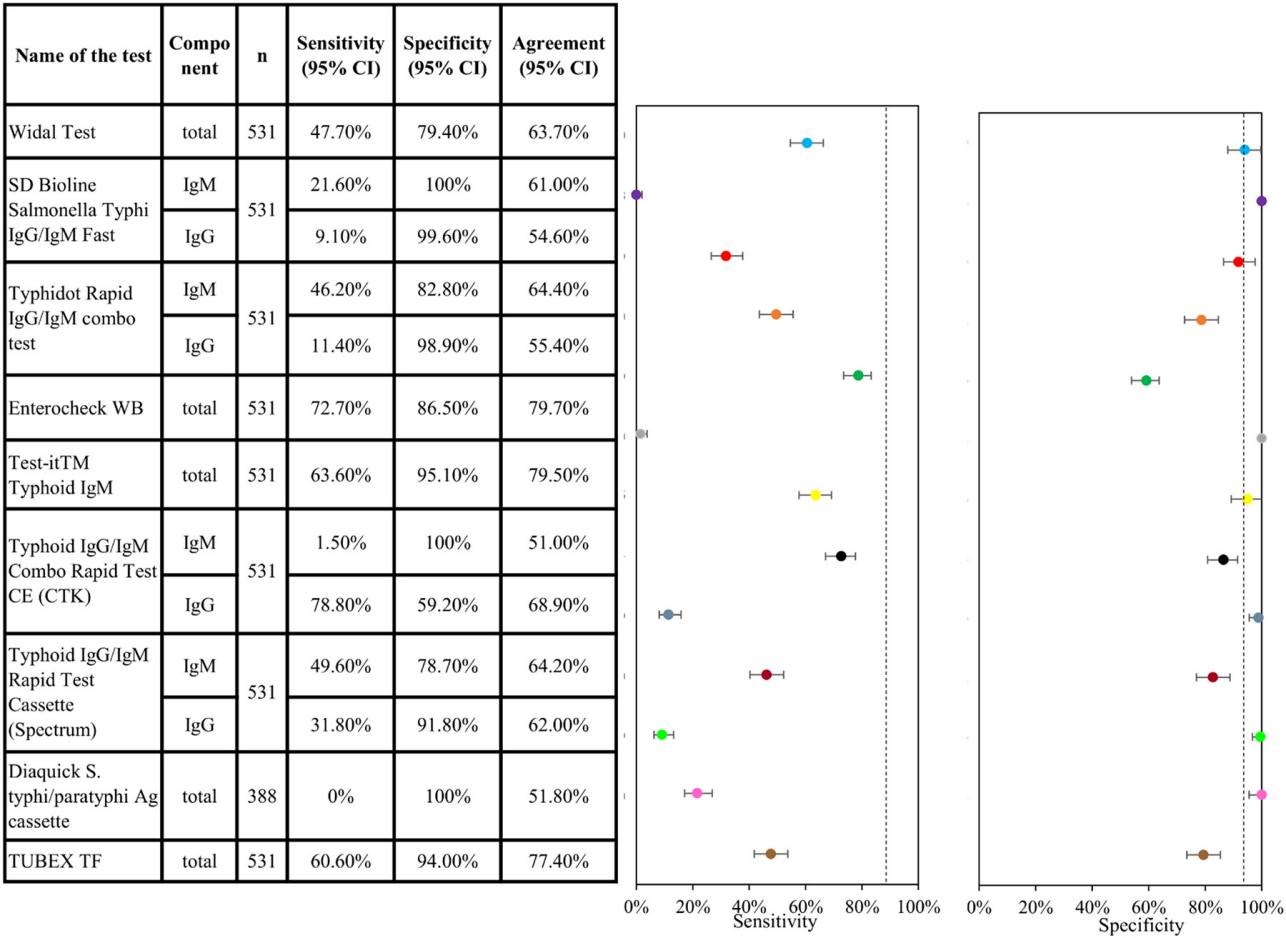
Sensitivity and specificity of nine S. Typhi RDTs (mITT population) assessed using blood culture testing to confirm diagnosis. CI, confidence interval (Wilson method); IgG, immunoglobulin G; IgM, immunoglobulin M; mITT, modified intent-to-test; RDT, rapid diagnostic test. Note that Diaquick S. Typhi IgG/IgM Ab tests are not done in the subjects of retrospective samples.

In total, 531 serum samples were evaluated using the nine RDTs (264 in positive cases and 267 in negative cases). The sensitivity and specificity of each RDT is summarized in Fig. 2. Sensitivity values varied widely between the different tests, from 0% (Diaquick Ag cassette) to 78.8% (IgG component of the Typhoid IgG/IgM Combo Rapid Test CE [CTK]). However, IgG tests show the presence of a past infection (14), and the sensitivity of the IgM component of the CTK test (for present infection) was very low (1.5%). The second highest sensitivity (72.7%) was observed with the Enterocheck WB IgM test (Tulip Diagnostics). The specificity values showed less variation between RDTs, ranging from 59.2% (IgG component of the Typhoid IgG/IgM Combo Rapid Test CE [CTK]) to 100% (Diaquick Ag Cassette, IgM components of the SD Bioline *S.* Typhi IgG/IgM Fast and Typhoid IgG/IgM Combo Rapid Test CE [CTK]). The agreement was calculated after the removal of invalid results by taking the proportion of samples detected as positive or negative by both index and reference tests over the total number of the sample.

**FIG 3:**
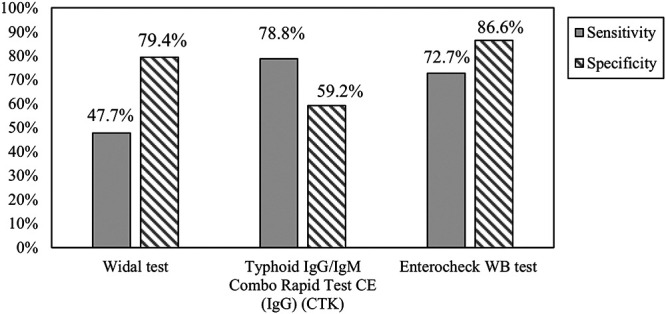
Comparison of Widal test with two best performing RDTs. IgG, immunoglobulin G; IgM, immunoglobulin M; RDT, S. Typhi rapid diagnostic test.

Overall, the sensitivity and specificity of RDTs was broadly similar (differences <20%) across both regions of endemicity with three exceptions (Table 2): the Typhoid IgG/IgM Rapid Test Cassette showed a greater sensitivity in Pakistan (IgG component: 58.0% versus 20.3%; [IgM component: 37.6% versus 11.9%]), the Test-it Typhoid IgM test and Typhidot Rapid IgG/IgM test (IgM component) showed a greater sensitivity in Kenya (81.4% versus 58.5 and 93.2% versus 32.7%, respectively; Table 2). Similar to the overall findings, the highest sensitivities were provided by the IgG component of the Typhoid IgG/IgM Combo Rapid Test CE (82.4% in Pakistan) and the Enterocheck WB IgM test (83.1% in Kenya). A comparison of the best-performing RDTs with the Widal test is presented in Fig. 3.

**TABLE 2:** Sensitivity and specificity of *S.* Typhi RDTs stratified by region of endemicity (Pakistan or Kenya [mITT population])

Test	Component	Pakistan (*n* = 410)			Kenya (*n* = 121)		
		No.	Sensitivity (95% CI)	Specificity (95% CI)	No.	% sensitivity (95% CI)	% specificity (95% CI)
Widal Test	Total	410	48.3 (41.5–55.1)	75.1 (68.8–80.5)	121	45.8 (33.7–58.3)	93.5 (84.6–97.5)
SD Bioline *S.* Typhi IgG/IgM Fast	IgM	410	19.5 (14.7–25.5)	100 (98.2–100)	121	28.8 (18.8–41.4)	100 (94.2–100)
	IgG	410	5.4(3.0–9.4)	99.5 (97.3–99.9)	121	22.0 (13.4–34.1)	100 (94.2–100)
Typhidot Rapid IgG/IgM Combo Test	IgM	410	32.7 (26.6–39.4)	93.2 (88.9–95.9)	121	93.2 (83.8–97.3)	48.4 (36.4–60.6)
	IgG	410	10.7 (7.2–15.7)	98.5 (95.8–99.5)	121	13.6 (7.0–24.5)	100 (94.2–100)
Enterocheck WB	Total	410	69.8 (63.2–75.6)	88.3 (83.2–92.0)	121	83.1 (71.5–90.5)	80.6 (69.1–88.6)
Test-it Typhoid IgM	Total	410	58.5 (51.7–65.1)	94.6 (90.6–97.0)	121	81.4 (69.6–89.3)	96.8 (89.0–99.1)
Typhoid IgG/IgM Combo Rapid Test CE (CTK)	IgM	410	0 (0.0–1.8)	100 (98.2–100)	121	6.8 (2.7–16.2)	100 (94.2–100)
	IgG	410	82.4 (76.6–87.0)	58.0 (51.2–64.6)	121	66.1 (53.4–76.9)	62.9 (50.5–73.8)
Typhoid IgG/IgM Rapid Test Cassette (Spectrum)	IgM	410	58.0 (51.2–64.6)	74.6 (68.3–80.1)	121	20.3 (12.0–32.3)	91.9 (82.5–96.5)
	IgG	410	37.6 (31.2–44.4)	89.8 (84.8–93.2)	121	11.9 (5.9–22.5)	98.4 (91.4–99.7)
Diaquick *S.* Typhi/Paratyphi Ag Cassette	Total	387	0 (0.0–2.0)	100 (98.1–100)	121	NP^*a*^	NP
TUBEX TF	Total	410	63.4 (56.6–69.7)	92.2 (87.7–95.1)	121	50.8 (38.4–63.2)	100 (94.2–100)

a NP, The Diaquick *S.* Typhi/paratyphi Ag cassette test was not performed in Kenya. CI, confidence interval; RDT, rapid diagnostic test; IgG, immunoglobulin G; IgM, immunoglobulin M; mITT, modified intent-to-test.

In latent class modeling, CTK IgG was found to have the highest sensitivity (79.1%), followed by Enterocheck (73.8%) (Table 3). The specificity ranged between 63.8% (CTK IgG) and 99.7% (Test-it). The highest overall accuracy was observed with Enterocheck, with 73.8% (95% CrI = 68.4 to 78.9%) sensitivity and 94.5% (95% CrI = 90.8 to 97.2%) specificity.

**TABLE 3:** Latent class modeling of the test results

Test	% Sensitivity			% Specificity		
	Median	95% CI		Median	95% CI	
		Min	Max		Min	Max
Blood culture	0.831	0.789	0.867	1	1	1
CTK IgG	0.791	0.739	0.83	0.638	0.582	0.701
Enterocheck	0.738	0.684	0.789	0.945	0.908	0.972
SD IgM	0.199	0.155	0.249	0.997	0.986	1
Spectrum IgM	0.497	0.44	0.552	0.821	0.77	0.863
TestIt	0.62	0.56	0.675	0.997	0.986	1
Tubex	0.582	0.529	0.639	0.971	0.943	0.989
Typhidot IgM	0.457	0.397	0.523	0.852	0.798	0.897
Widal	0.486	0.425	0.545	0.837	0.784	0.881
Prevalence	0.548	0.504	0.591			

### Secondary outcomes.

A biorepository of 389 characterized serum samples (and basic clinical data) was established at the Aga Khan University central laboratory (187 typhoid positive and 202 typhoid negative) for use in the development of new tests and the evaluation of emerging technologies in the future via the FIND biobank (https://www.finddx.org/biobank-services/specimen-bank/mrf/). Invalid tests were reported for two of the RDTs, the Test-it Typhoid IgM test (0.9% invalid test rate) and the Diaquick Ag cassette (23.8% invalid test rate).

## DISCUSSION

This study evaluated the performance of nine commercially available S. Typhi RDTs in two geographically distant regions of endemicity using a single, established test as a reference standard (blood culture). In general, the RDTs showed poor sensitivity (0 to 79%) compared to the rigorous standards set by World Health Organization (WHO) for an effective laboratory test for the diagnosis of typhoid (15). The specificity of the RDTs was higher but still not always >90%. In particular, the Widal test showed very low sensitivity and specificity (47.7 and 79.4%, respectively) and the SD Bioline *S.* Typhi IgG/IgM Fast test showed very low sensitivity (21.6% for IgM, 9.1% for IgG) but nearly 100% specificity. For most of the tests, the difference between regions was minimal, with only the IgM component of Typhoid IgG/IgM Rapid Test Cassette (spectrum), the Test-it Typhoid IgM test, and the IgM component of Typhidot Rapid IgG/IgM showing >20% differences in sensitivity (one greater in Pakistan, later two greater in Kenya). Overall, the best-performing RDT was the Enterocheck WB, with 72.7% sensitivity and 86.5% specificity. While the Typhoid IgG/IgM Combo Rapid Test CE showed a higher sensitivity for IgG, this typically reflects a previous infection (14), and the sensitivity for acute infection (IgM) was very low.

In general, the findings of this study are supported by those of previous studies (16–24). A study in Bangladesh reported similar findings for the SD Bioline *S*. Typhi IgG/IgM Fast, the Typhoid IgG/IgM Combo Rapid Test CE (IgM component) and Test-it Typhoid IgM (16). The specificity findings for the Typhidot Rapid IgG/IgM Combo test are also consistent with a recent study conducted in Fiji, as are the Test-it Typhoid IgM findings (17). The sensitivity and/or specificity results for the TUBEX TF test were consistent with those reported in studies conducted in various regions, including Bangladesh, Southern Vietnam and Papua New Guinea, as well as a systematic review and meta-analysis of studies in several countries where typhoid is endemic (9, 18, 19, 21). Finally, the poor sensitivity and specificity showed by the Widal test in this study is in line with established consensus and the findings from many previous studies (6, 8, 9, 22–24).

However, there are also studies for many of the RDTs that show slightly to widely contrasting sensitivity and specificity values (16–18, 25–28), probably due to smaller sample sizes and reference standard differences. Nevertheless, considering the head-to-head nature of this study and the consistent methodology/reference standards employed, together with the broad consensus of the findings across the literature, the results presented here may help the WHO and health provider decision-making regarding the utility of commercial RDTs in different settings and use cases, while clearly highlighting which tests should not be used in practice. The findings further highlight the critical investment in the development of next-generation RDTs that could be used at the point of care.

Limitations to this study include the use of blood culture as a reference standard, which itself is an imperfect test with limited sensitivity (6). Alternatives to blood culture testing also have their challenges, with bone marrow testing being a complicated and invasive procedure and PCR testing also having limited sensitivity (6). Currently, there is no simple, well-performing, accessible test to use as a gold-standard reference standard. To address this, we used Bayesian latent class modeling to jointly estimate the accuracy of RDTs, using their mutual information and accounting for the imperfect sensitivity of blood culture to better estimate true diagnostic accuracy. This approach has been used in several previous typhoid diagnostics studies (29–31). Another limitation is that RDTs were performed by laboratory staff who had been specifically trained in the appropriate procedures. In routine clinical practice, users would likely follow the manufacturer’s instructions without specialized training, which could potentially mean that differences in RDT ease of use could result in differences in the test results (e.g., invalid test rates). Future real-world evidence studies may provide further insight. The prevalence of S. Typhi was very low in Kenya than the expected which may be due to strict social restriction for COVID-19 pandemics during data collection whereas in Pakistan there was no total lockdown observed during study period. The lack of prospective samples was addressed by inclusion of the retrospective samples. However, the limited number of retrospective samples in the study precluded comparisons between prospective and retrospective samples to detect any differences (e.g., as a result of Ig deterioration in stored samples). The use of frozen retrospective samples in one of the study sites might have impacted the performance of the RDTs. However, this is unlikely since, apart from the IgM component of the Typhidot Rapid IgG/IgM, all other test results were comparable between sites. Retesting of the samples for incomparable results was not possible due to the lack of samples in the retrospective study. Finally, the RDTs here were performed only after blood culture results were available in a limited number of participants rather than when patients presented with fever, but the sample size included in the study allowed us to estimate the sensitivity of the evaluated RDTs with 80% power to detect the 95% CI.

In conclusion, all of the S. Typhi RDTs evaluated in this study had sensitivity and specificity values that fell below the required levels to be recommended for an accurate diagnosis. However, the Enterocheck WB and Test-it Typhoid IgM tests performed best and were considerably better than the widely used Widal test, despite not being commonly used in clinical practice. There were minimal differences in RDT performances between regions of endemicity, with only the Typhoid IgG/IgM Rapid Test Cassette and the Test-it Typhoid IgM test showing >20% differences in sensitivity.

Overall, the performance of the S. Typhi RDTs in this study highlights the clear need for new developments in the typhoid test landscape, and it is hoped the biorepository of characterized serum samples established at the Aga Khan University central laboratory will help in the development and evaluation of this next generation of tests.

## References

[1] Crump JA. 2019. Progress in typhoid fever epidemiology. Clin Infect Dis 68:S4–S9. doi:10.1093/cid/ciy846. 30767000 PMC6376096

[2] Amicizia D, Micale RT, Pennati BM, Zangrillo F, Iovine M, Lecini E, Marchini F, Lai PL, Panatto D. 2019. Burden of typhoid fever and cholera: similarities and differences: prevention strategies for European travelers to endemic/epidemic areas. J Prev Med Hyg 60:E271–E285. 31967084 10.15167/2421-4248/jpmh2019.60.4.1333 PMC6953460

[3] Ayukekbong JA, Ntemgwa M, Atabe AN. 2017. The threat of antimicrobial resistance in developing countries: causes and control strategies. Antimicrob Resist Infect Control 6:47. doi:10.1186/s13756-017-0208-x. 28515903 PMC5433038

[4] Meiring JE, Giubilini A, Savulescu J, Pitzer VE, Pollard AJ. 2019. Generating the evidence for typhoid vaccine introduction: considerations for global disease burden estimates and vaccine testing through human challenge. Clin Infect Dis 69:S402–S407. doi:10.1093/cid/ciz630. 31612941 PMC6792111

[5] Hamaguchi S, Cuong NC, Tra DT, Doan YH, Shimizu K, Tuan NQ, Yoshida L-M, Mai LQ, Duc-Anh D, Ando S, Arikawa J, Parry CM, Ariyoshi K, Thuy PT. 2015. Clinical and epidemiological characteristics of scrub typhus and murine typhus among hospitalized patients with acute undifferentiated fever in Northern Vietnam. Am J Trop Med Hyg 92:972–978. doi:10.4269/ajtmh.14-0806. 25778504 PMC4426587

[6] Mather RG, Hopkins H, Parry CM, Dittrich S. 2019. Redefining typhoid diagnosis: what would an improved test need to look like? BMJ Glob Health 4:e001831. doi:10.1136/bmjgh-2019-001831. PMC6830052 31749999

[7] Moore CE, Pan-Ngum W, Wijedoru LPM, Sona S, Nga TVT, Duy PT, Vinh PV, Chheng K, Kumar V, Emary K, Carter M, White L, Baker S, Day NPJ, Parry CM. 2014. Evaluation of the diagnostic accuracy of a typhoid IgM flow assay for the diagnosis of typhoid fever in Cambodian children using a Bayesian latent class model assuming an imperfect gold standard. Am J Trop Med Hyg 90:114–120. doi:10.4269/ajtmh.13-0384. 24218407 PMC3886406

[8] Khanam F, Sheikh A, Sayeed M, Bhuiyan M, Choudhury FK, Salma U, Pervin S, Sultana T, Ahmed D, Goswami D, Hossain M, Mamun KZ, Charles RC, Brooks WA, Calderwood SB, Cravioto A, Ryan ET, Qadri F. 2013. Evaluation of a typhoid/paratyphoid diagnostic assay (TPTest) detecting anti-*Salmonella* IgA in secretions of peripheral blood lymphocytes in patients in Dhaka, Bangladesh. PLoS Negl Trop Dis 7:e2316. doi:10.1371/journal.pntd.0002316. 23951368 PMC3708850

[9] Siba V, Horwood PF, Vanuga K, Wapling J, Sehuko R, Siba PM, Greenhill AR. 2012. Evaluation of serological diagnostic tests for typhoid fever in Papua New Guinea using a composite reference standard. Clin Vaccine Immunol 19:1833–1837. doi:10.1128/CVI.00380-12. 22993409 PMC3491554

[10] Basnyat B. 2016. Typhoid versus typhus fever in post-earthquake Nepal. Lancet Glob Health 4:e516–e517. doi:10.1016/S2214-109X(16)30094-8. 27443773

[11] World Health Organization. 1999. WHO recommended surveillance standards. WHO/CDS/CSR/ISR/99.2. World Health Organization, Geneva, Switzerland.

[12] Saha SK, Baqui AH, Hanif M, Darmstadt GL, Ruhulamin M, Nagatake T, Santosham M, Black RE. 2001. Typhoid fever in Bangladesh: implications for vaccination policy. Pediatr Infect Dis J 20:521–524. doi:10.1097/00006454-200105000-00010. 11368111

[13] Joseph L, Gyorkos TW, Coupal L. 1995. Bayesian estimation of disease prevalence and the parameters of diagnostic tests in the absence of a gold standard. Am J Epidemiol 141:263–272. doi:10.1093/oxfordjournals.aje.a117428. 7840100

[14] Qudsia F, Rehan M, Riaz S. 2020. Molecular analysis of immunoglobulins related to salmonella typhi in pediatric patients. Arch Pathol Clin Res 4:5–10.

[15] Baker S, Favorov M, Dougan G. 2010. Searching for the elusive typhoid diagnostic. BMC Infect Dis 10:45. doi:10.1186/1471-2334-10-45. 20205702 PMC2846943

[16] Maude RR, de Jong HK, Wijedoru L, Fukushima M, Ghose A, Samad R, Hossain MA, Karim MR, Faiz MA, Parry CM, CMCH Typhoid Study Group. 2015. The diagnostic accuracy of three rapid diagnostic tests for typhoid fever at Chittagong Medical College Hospital, Chittagong, Bangladesh. Trop Med Int Health 20:1376–1384. doi:10.1111/tmi.12559. 26094960 PMC4832346

[17] Getahun Strobel A, Airs S, Nguyen C, Vadei TR, Matanitobua S, Kama M, Watson CH, Crump JA, Mulholland EK, Strugnell RA, Parry CM. 2021. Assessment of rapid diagnostic tests for typhoid diagnosis and assessment of febrile illness outbreaks in Fiji. Am J Trop Med Hyg 106:543–549. doi:10.4269/ajtmh.21-0771. 34844208 PMC8832939

[18] Naheed A, Ram PK, Brooks WA, Mintz ED, Hossain MA, Parsons MM, Luby SP, Breiman RF. 2008. Clinical value of Tubex and Typhidot rapid diagnostic tests for typhoid fever in an urban community clinic in Bangladesh. Diagn Microbiol Infect Dis 61:381–386. doi:10.1016/j.diagmicrobio.2008.03.018. 18501549

[19] Olsen SJ, Pruckler J, Bibb W, Thanh NTM, Trinh TM, Minh NT, Sivapalasingam S, Gupta A, Phuong PT, Chinh NT, Chau NV, Cam PD, Mintz ED. 2004. Evaluation of rapid diagnostic tests for typhoid fever. J Clin Microbiol 42:1885–1889. doi:10.1128/JCM.42.5.1885-1889.2004. 15131144 PMC404619

[20] Tarupiwa A, Tapera S, Mtapuri-Zinyowera S, Gumbo P, Ruhanya V, Gudza-Mugabe M, Majuru NX, Chin’ombe N. 2015. Evaluation of TUBEX-TF and OnSite Typhoid IgG/IgM Combo rapid tests to detect *Salmonella enterica* serovar Typhi infection during a typhoid outbreak in Harare, Zimbabwe. BMC Res Notes 8:50. doi:10.1186/s13104-015-1015-1. 25890321 PMC4344803

[21] Thriemer K, Ley B, Menten J, Jacobs J, van den Ende J. 2013. A systematic review and meta-analysis of the performance of two point of care typhoid fever tests, Tubex TF and Typhidot, in endemic countries. PLoS One 8:e81263. doi:10.1371/journal.pone.0081263. 24358109 PMC3864786

[22] Bhutta ZA, Mansurali N. 1999. Rapid serologic diagnosis of pediatric typhoid fever in an endemic area: a prospective comparative evaluation of two dot-enzyme immunoassays and the Widal test. Am J Trop Med Hyg 61:654–657. doi:10.4269/ajtmh.1999.61.654. 10548305

[23] Hoffman SL, Flanigan TP, Klaucke D, Leksana B, Rockhill RC, Punjabi NH, Pulungsih SP, Sutomo A, Moechtar MA. 1986. The Widal slide agglutination test, a valuable rapid diagnostic test in typhoid fever patients at the Infectious Diseases Hospital of Jakarta. Am J Epidemiol 123:869–875. doi:10.1093/oxfordjournals.aje.a114316. 3962968

[24] Ohanu ME, Iroezindu MO, Maduakor U, Onodugo OD, Gugnani HC. 2019. Typhoid fever among febrile Nigerian patients: prevalence, diagnostic performance of the Widal test and antibiotic multidrug resistance. Malawi Med J 31:184–192. doi:10.4314/mmj.v31i3.4. 31839887 PMC6895380

[25] Kawano RL, Leano SA, Agdamag DMA. 2007. Comparison of serological test kits for diagnosis of typhoid fever in the Philippines. J Clin Microbiol 45:246–247. doi:10.1128/JCM.01403-06. 17065261 PMC1828988

[26] Bhume RJ, Babaliche P. 2020. Clinical profile and the role of rapid serological tests: Typhifast IgM and Enterocheck WB in the diagnosis of typhoid fever. Indian J Crit Care Med 24:307–312. doi:10.5005/jp-journals-10071-23417. 32728320 PMC7358864

[27] Keddy KH, Sooka A, Letsoalo ME, Hoyland G, Chaignat CL, Morrissey AB, Crump JA. 2011. Sensitivity and specificity of typhoid fever rapid antibody tests for laboratory diagnosis at two sub-Saharan African sites. Bull World Health Organ 89:640–647. doi:10.2471/BLT.11.087627. 21897484 PMC3165980

[28] Mawazo A, Bwire GM, Matee MIN. 2019. Performance of Widal test and stool culture in the diagnosis of typhoid fever among suspected patients in Dar es Salaam, Tanzania. BMC Res Notes 12:316. doi:10.1186/s13104-019-4340-y. 31167646 PMC6551910

[29] Andrews JR, Khanam F, Rahman N, Hossain M, Bogoch II, Vaidya K, Kelly M, Calderwood SB, Bhuiyan TR, Ryan ET, Qadri F, Charles RC. 2019. Plasma immunoglobulin a responses against 2 *Salmonella* Typhi antigens identify patients with typhoid fever. Clin Infect Dis 68:949–955. doi:10.1093/cid/ciy578. 30020426 PMC6399438

[30] Islam K, Sayeed MA, Hossen E, Khanam F, Charles RC, Andrews J, Ryan ET, Qadri F. 2016. Comparison of the performance of the TPTest, Tubex, Typhidot and Widal immunodiagnostic assays and blood cultures in detecting patients with typhoid fever in Bangladesh, including using a Bayesian latent class modeling approach. PLoS Negl Trop Dis 10:e0004558. doi:10.1371/journal.pntd.0004558. 27058877 PMC4825986

[31] Arora P, Thorlund K, Brenner DR, Andrews JR. 2019. Comparative accuracy of typhoid diagnostic tools: a Bayesian latent-class network analysis. PLoS Negl Trop Dis 13:e0007303. doi:10.1371/journal.pntd.0007303. 31067228 PMC6527309

