# Supplementary material for: Comparative Analysis of Commercially Available Typhoid Point-of-Care Tests: Results of a Prospective and Hybrid Retrospective Multicenter Diagnostic Accuracy Study in Kenya and Pakistan
Source: J Clin Microbiol. 2022 Nov 30;60(12):e01000-22. doi: 10.1128/jcm.01000-22 (PMC9769786; doi:10.1128/jcm.01000-22)
Supplement: Supplemental file 1 — Table S1 and Fig. S1. Download jcm.01000-22-s0001.pdf, PDF file, 0.01 MB [file jcm.01000-22-s0001.pdf]

S. no	Assay/System	Assay type	Target	Volume of sample (Serum)	Type of sample	Time to result
1	Widal Test	Slide Agglutination	O and H antibodies	80µl	Serum	15 mins
2	SD BIOLINE SALMONELLA TYPHI IgG/IgM FAST (Abbott)	Dipstick	IgG/IgM	1µl	Serum/Plasma/ WB	15-30 mins
3	Typhidot Rapid IgG/IgM combo test (Reszon Diagnostica)	Lateral Flow assay	IgG/IgM	90µl	Serum/Plasma	20 mins
4	Enterocheck WB (Tulip Diagnostics)	Lateral Flow assay	IgM	5µl	Serum/Plasma/ WB	15 mins
5	Test-it™ Typhoid IgM (Life Asssay)	Lateral Flow assay	IgM	5µl	Serum/WB	15mins
6	Typhoid IgG/IgM Combo Rapid Test CE (CTK Biotech)	Lateral Flow assay	IgG/IgM	40µl	Serum/Plasma/ WB	15 mins
7	Typhoid IgG/IgM Rapid Test Casette (Spectrum Diagnostics))	Lateral Flow assay	IgG/IgM	45µl	Serum/Plasma	15mins
8	Diaquick S. typhi/paratyphi Ag cassette (Dialab)	Lateral Flow assay	Ag	120µl	Serum/Plasma/ WB/stool	20mins
9	TUBEX TF (IDL Biotech)	Inhibition Magnetic binding Immunoassay-Colorometric assay	IgM	45µl	Serum/Plasma	10mins

Supplementary table 1: Characteristics of Typhoid RDTs included in the evaluation

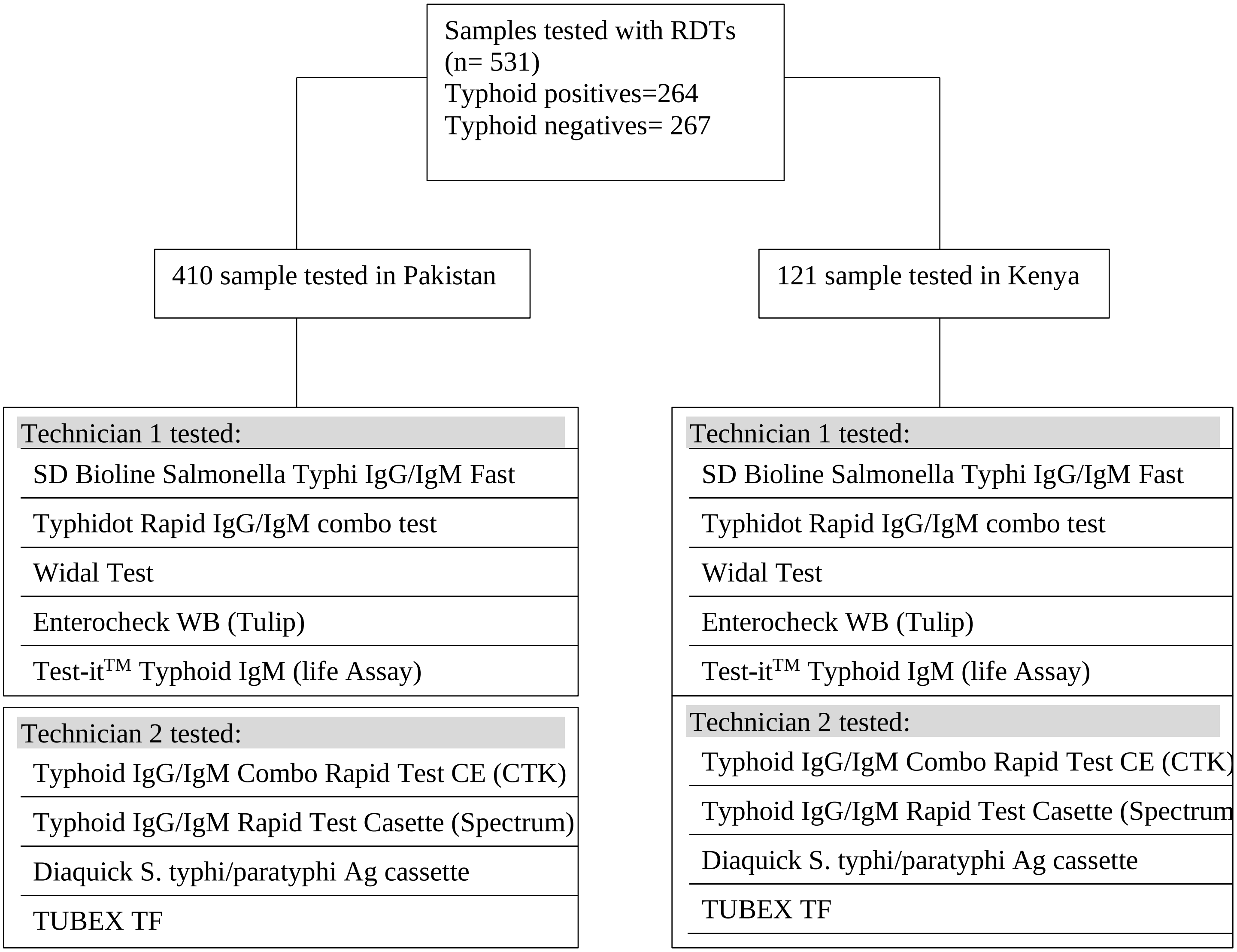

Supplementary Figure 1: RDTs testing Flow diagram:
Note: Technicians performing RDTs were blinded of blood culture report.
